# Spinal Fusion in the Next Generation: Gene and Cell Therapy Approaches

**DOI:** 10.1155/2014/406159

**Published:** 2014-01-28

**Authors:** Marta Barba, Claudia Cicione, Camilla Bernardini, Vincenzo Campana, Ernesto Pagano, Fabrizio Michetti, Giandomenico Logroscino, Wanda Lattanzi

**Affiliations:** ^1^Institute of Anatomy and Cell Biology, Università Cattolica del Sacro Cuore, Largo Francesco Vito, 1, 00168 Rome, Italy; ^2^Departement of Orthopaedics and Traumatology, Università Cattolica del Sacro Cuore, Largo Agostino Gemelli, 8, 00168 Rome, Italy; ^3^Latium Musculoskeletal Tissue Bank, Largo Francesco Vito, 1, 00168 Rome, Italy

## Abstract

Bone fusion represents a challenge in the orthopedics practice, being especially indicated for spine disorders. Spinal fusion can be defined as the bony union between two vertebral bodies obtained through the surgical introduction of an osteoconductive, osteoinductive, and osteogenic compound. Autogenous bone graft provides all these three qualities and is considered the gold standard. However, a high morbidity is associated with the harvest procedure. Intensive research efforts have been spent during the last decades to develop new approaches and technologies for successful spine fusion. In recent years, cell and gene therapies have attracted great interest from the scientific community. The improved knowledge of both mesenchymal stem cell biology and osteogenic molecules allowed their use in regenerative medicine, representing attractive approaches to achieve bone regeneration also in spinal surgery applications. In this review we aim to describe the developing gene- and cell-based bone regenerative approaches as promising future trends in spine fusion.

## 1. Introduction

Spine fusion is a surgical technique used to join two or more vertebrae and to stabilize the corresponding spine segment. It is frequently used to treat traumatic and degenerative spine disease, such as scoliosis, kyphosis, fractures, dislocations, spondylolisthesis, and intervertebral disc diseases [1–3].

The fusion is achieved through stabilization systems adding supplementary bone tissue and/or bone substitutes between adjacent vertebrae, as to enhance bone healing and to achieve faster stability. Three types of bone grafts can be classified (Table 1): (i) autografts: the donor is the same as the receiver; (ii) allografts: the donor is human but is different from the receiver; (iii) xenografts: the donor is from different animal species (heterologous graft).

The process of spinal fusion requires three essential characteristics: osteoconductivity, osteoinductivity, and osteogenicity. Autologous bone graft has all these properties, provides an ideal material for spine fusion, and has long been considered the gold standard for fusion procedures. A significant morbidity is inherently associated with the harvest procedure, as a bone defect is created, requiring also prolonged surgical duration [4, 5]. Moreover, the limited availability of autologous bone is a significant limitation.

Allograft bone is routinely used as an alternative to autogenous bone to avoid complications related to donor site morbidity and availability. Nonetheless, concerns about immunogenicity and infectious disease transmission are ascribed to its use [6, 7].

Xenografts represent an alternative strategy, employed more frequently in dental surgery than in orthopedic surgery. In theory, the principal disadvantage of heterologous graft is the high level of antigenicity. Partially deproteinated and partially defatted heterologous bone (Kiel bone or Oswestry bone) exhibits a significantly reduced antigenicity and minimal immune response, but the denaturation process destroys the matrix proteins, damaging the osteoinductive properties. Also, the risk of zoonoses for diseases such as BSE (bovine spongiform encephalopathy) or PERV (porcine endogenous retroviruses) has been often discussed [8].

These and other difficulties with bone grafts have been driving the intensive research efforts that have been spent during the last decades to develop new approaches and technologies for successful spine fusion. On this regard, synthetic bone substitutes have been proposed as valuable alternative options (Table 1), based on osteoconductive/osteoinductive biomaterials owing the ability (i) to generate a microenvironment which induces the cellular growth; (ii) to recruit bone precursor cells (osteoconductivity) in the area surrounding the implant site,; (iii) to induce cell proliferation and differentiation required for the osteogenic process (osteoinductivity) [9]. New calcium and phosphate-based substitutes have been developed, leading to the generation of biomaterials known as “bioceramics.” Often a mixture of hydroxyapatite (HA) and its amorphous phase, the tricalcium phosphate (TCP), is used to obtain bioactive ceramics, which form direct chemical bonds with bone or even with soft tissues of a living organism [10, 11].

In recent years, cell and gene therapies have attracted great interest from the scientific community and have shown to represent promising approaches to achieve bone regeneration also in spine surgery. The improved knowledge on adult stem cell biology and of mesenchymal stem cell features allowed their use in regenerative medicine, with particular focus on bone regeneration. In particular, cell-based approaches based on mesenchymal stem cells (MSC) have been widely employed and considered the most effective for bone formation and regeneration *in vivo *[12, 13]. In addition, the overwhelming amount of studies that have been investigating the molecular scenario orchestrating osteogenesis and bone healing, provided new osteoinductive molecules to be tested as potential drugs in spine surgery. On the other hand, cell-based gene therapy approaches based on engineered-osteoinductive cells allowed achieving the most convincing results in terms of bone healing and spine fusion in animal models [14–19]. Actually, genetically engineered cells are believed to maintain physiologic doses of a gene product for a sustained period once inoculated into the selected anatomical site, facilitating an efficient bone healing [20].

Taken together, the developing molecular and cell-based bone regenerative approaches may plausibly represent promising future trends in spine fusion and will be reviewed below in detail.

## 2. Cell-Therapies for Spinal Fusion

Mesenchymal stem cells (MSCs) have been widely used as suitable somatic cells to induce bone formation and regeneration. MSCs are multipotent stem cells that are capable of extensive self-renewal, plasticity, and multilineage potential [21, 22]. These cells are located in the connective stroma of mesenchymal-derived adult organs and tissues; hence they are also named “stromal stem cells” [23]. Strictly defined MSCs are those isolated from bone marrow aspirates (bone marrow-mesenchymal stem cells, BM-MSCs); though, cells displaying high similarities have been found practically in quite any organ comprising a connective stroma including adipose tissue, lung, skeletal muscle, synovial membrane, tendons, and skin, along with antenatal tissues such as umbilical cord, placenta, and amniotic fluid [22, 24–30]. MSCs are easily isolated through adherence selection *in vitro* and can be further cultured for several passages, without losing their plasticity and self-renewal potential [31]. Upon appropriate *in vitro* induction, MSCs can be differentiated along the osteogenic lineage [32]. This property has been exploited for cell-based therapy of congenital bone disorders [33, 34]. The feasibility of an MSC-therapy for orthopedic disorders comes also from their immunomodulatory properties, implying their potential use in allogeneic transplantation, preventing graft-versus-host disease [35].

**Figure 1:**
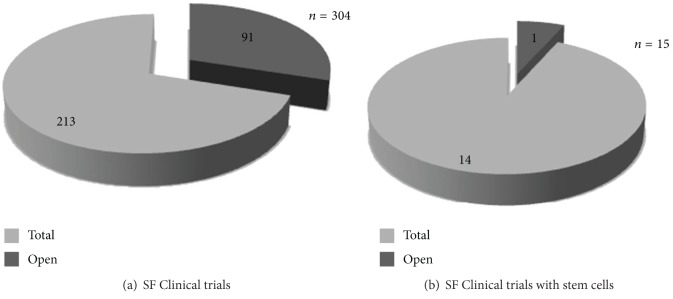
Spinal fusion clinical trials. Graphical view of the 304 clinical trials from http://www.clinicaltrials.gov/; 91 of these are open (a). In particular, spinal fusion clinical trials based on stem cell-therapy are 15; 1 of these is open (b).

A single clinical trial testing *ex vivo* expanded autologous BM-MSC for spinal fusion in spine degenerative diseases seems to be open, according to publicly available information (Figure 1; http://www.clinicaltrials.gov/).

In the clinical practice, BM-MSCs are usually harvested from the iliac crest (IC), through an invasive and painful procedure. To limit such donor morbidity issues, vertebral body (VB) bone marrow has been proposed as an alternative source of BM-MSCs. Compared to IC, VB-MSCs are easily isolated from the surgical site and show a higher amount of osteoprogenitor cells [36, 37]. However, both sources are subjected to a significant decline in stem cell number and proliferative capacities in elderly, when main indications for regenerative medicine approaches are encountered [38]. In addition, the number of MSC in bone marrow is reported to be 1 out of 5000 (0.0002%) total isolated cells [39]. Recently, adipose tissue has been highlighted as an excellent source of MSCs (namely adipose derived stem cells, ASCs) [40]. In particular, the adipose stromal vascular fraction (SVF) has emerged as a rich and promising source of ASCs [40], displaying extensive plasticity and multilineage differentiation potential [24, 41–44]. BM-MSCs and ASCs share part of their immunophenotype and gene expression profile, which is consistent with their stemness upholding and uncommitted [45–47]. Potentially, great advantages of ASCs over BM-MSCs are suggested by the high plasticity and extended self-renewal capability of these cells and by the abundance of adipose tissue, its surgical accessibility, and its high cellular content. Additionally, adipose tissue is now considered the largest human endocrine organ due to its role in the regulation of cellular functions, through a complex network of endocrine, paracrine, and autocrine signals [48]. There is actually an intense cross-talk between bone and adipose tissue, mediated by proteins endowed with endocrine functions secreted by adipocytes (adipokines) and osteoblasts (osteokines), which may suggest the feasibility of the use of SVF in bone regeneration, also due to the high angiogenicity endowed with the SVF [49, 50].

Distinct preclinical studies have tested the effectiveness of the MSCs from different tissue sources, in animal models of spinal fusion, combined with alternative scaffolds, with alternative scaffold, with successful results (Table 2). Most studies transplanted allogeneic cells into immunocompetent recipient animals [51–57].

Several animal studies used either wild type or osteogenic-committed BM-MSCs as possible substitutes of autologous bone-graft, with a good rate of spinal fusion [51–53, 55, 57–63]. The higher rate of spinal fusion has been obtained with culture-expanded BM-MSCs compared to freshly isolated cells [64]. In addition, Nakajima and colleagues [65] assessed that osteodifferentiated MSCs were more efficient in promoting spinal fusion than undifferentiated cells.

Interestingly, ASCs proved to allow bone regeneration *in vivo*, without the need for *ex vivo* engineering and/or induction [12]. Also, it has been clearly demonstrated that allogeneic ASCs displays a nonimmunogenic profile *in vitro* and does not evoke cell-based immunity when implanted in a rat spinal fusion model [66]. Taken together, these data could provide quite convincing proof-of-principle on the potential safeness and efficacy of banked MSCs from healthy donors. Though, the required standards for clinical-grade cell manufacturing (i.e., the current good manufacturing practices, cGMP, guidelines) would be quite hard to be met by the current experimental protocols employed for ASC isolation and culture [67]. However, these problems can be overcome with the development of single-step procedures to treat spinal disorders, by combining freshly harvested SVF and scaffolds [68, 69].

Besides MSC, fibroblasts have been proposed as suitable cell types for bone regenerative purposes. In particular, dermal fibroblasts (DF) can be easily isolated from small skin biopsies, with reduced local morbidity, and rapidly expanded in culture. Dermal fibroblasts share significant similarities with MSCs, being considered the skin-derived counterpart, and can be induced rapidly towards the osteogenic lineage [20, 70, 71]. Such features render DF a potentially promising tool for bone formation and regeneration.

## 3. Gene Therapies for Spinal Fusion

Gene therapy approaches are based on the rationale of delivering osteoinductive genes locally to induce bone formation and improve spinal fusion [15, 20, 72].

Different gene strategies have been proposed and tested as innovative strategies in spinal surgery to increase the osteogenic potential of osteoprogenitor cells and to obtain a higher bone formation rate *in vivo*. Bone morphogenetic proteins (BMP) represent the best characterized molecules implicated in the osteogenic cascade and have been widely employed to induce bone formation in spinal fusion models [73].

The BMP family is composed of 20 distinct highly conserved secreted proteins, further categorized into multiple subgroups according to functional and/or structural features [90, 91]. BMP play a pivotal role in skeletogenesis during limb development processes. In particular, they increase osteoclastogenesis and induce the osteoblastic commitment of MSC, inhibiting their differentiation along the myoblastic and adipogenic lineages [91–94]. The osteogenic BMP, are BMP2, BMP4 and BMP7 (also known as osteogenic protein-1, OP-1). They can induce the differentiation of MSC into both osteochondrogenic lineage cells and osteoblast precursor cells, implicating their essential contribution to both direct and indirect ossification mechanisms occurring in vertebrates [95–97]. A wide number of preclinical studies have demonstrated that these small molecules are capable of inducing ectopic bone formation upon intramuscular implantation and efficient bone healing/regeneration, when delivered on the appropriate scaffold and in the appropriate concentration into a bone defect site [12, 14, 98]. In addition, the use of recombinant human BMP2 (rhBMP2) and BMP7 (rhBMP7) has been approved in both Europe and the United States for selected clinical applications, including lumbar interbody spinal fusion and tibial non-union defects. Nowadays, various genetic engineering approaches are being considered to produce second-generation BMPs, aimed at improving binding affinity to target specific cells, reducing sensitivity to natural inhibitors, reducing immunogenicity, and increasing solubility and stability [99].

The main limitation of using recombinant proteins for inducing bone formation in clinical applications is the need for delivery systems that provide a sustained and biologically appropriate concentration of the osteogenic factor at the site of the defect [12, 20]. The vectors used for gene therapy approaches comprise naked DNA, liposomes, plasmids, and viral vectors [100]. Nonviral vectors are generally safer due to the absence of infectious-related issues. However, they have a low transfection efficiency. The viral vector commonly used in gene therapy approaches for spinal fusion belongs to the adenovirus species. Defective human adenoviruses are indeed suitable gene vectors due to their ability to mediate high-level and short-term gene expression. Although their use implies several disadvantages in view of a potential clinical application [14], adenoviral vectors carrying osteoinductive genes have been successfully used in preclinical spinal fusion models (Table 2). Most studies employed defective adenoviral vectors carrying the BMP2 gene (AdBMP2), either for *ex vivo* cell transduction [54, 70, 78, 79, 87] or for direct percutaneous injection [101]. The chance of spinal fusion increases by using cells for achieving an appropriate local gene delivery. In fact, genetically engineered cells can mediate the local expression of osteoinductive genes in a time- and site-effective manner, thus mimicking the physiologic secretion* in vivo*. Engineered cell-based therapy approaches resulted in being indeed more efficient than recombinant osteoinductive proteins alone [102, 103]. However, despite the significant evidence of their potential benefit to bone repair, there is, to date, a dearth of convincing clinical trials [104].

With regard to the cell type, Miyazaki and colleagues recently demonstrated that the efficacy of AdBMP2-transduced MSC treatment is not related to the tissue source for cell isolation. ASCs and BM-MSCs proved to exert comparable results in a rat spinal fusion model [105]. Adenoviral vectors carrying BMP4 [106], BMP6 [107], and BMP9 [105] were also used for direct injection into the paraspinal musculature, which proved to be effective. Also BMP7 has been tested as a suitable molecule delivered *ex vivo* in BM-MSC to induce spine fusion [80].

Nonetheless, several contraindications hinder the use of adenoviral vectors in humans, including systemic toxicity, immunization (over 95% adults have neutralizing antibodies against adenovirus species 5), and low cell selectivity [14].

A lentivirus-based BMP2 vector (lenti-BMP2) has been also tested as a feasible tool to induce stable osteogenic commitment of BM-MSCs in a rat spinal fusion model [81]. Lenti-BMP2 is a specialized retrovirus capable of random integration in the host cell genome. This strategy proved to be more effective than AdBMP2-based cell transduction [54]. However, the possible risks of insertional mutagenesis should be carefully considered when using lentiviral vectors [14].

Recently, a nonviral approach was attempted using nucleofection (i.e., the intranuclear transfection by electroporation) of rhBMP6 in ASCs [89]. The results obtained through this virus-free technology sound encouraging, although the plasmid DNA used in the procedure still retains some inherent bacterial-related toxicity.

Besides BMP, other molecules have been tested for their osteogenic potential in gene therapy approaches for spinal fusion. These included the Nell-like molecule (Nell-1) [108, 109], the LIM mineralization protein (LMP) [20, 110], and the mothers against decapentaplegic homolog 1 (Smad1) [83].

Nell-1 is a heterotrimeric secretory protein thought to be involved in cell growth regulation and differentiation, acting specifically in osteoblasts. Nell-1 is overexpressed in synostotic calvaria of patients affected by sporadic plagiocephaly [111] and is able to induce bone regeneration in rat calvarial defects [112]. Based on the evidence that this gene is more osteoblast specific than BMP, the efficacy of AdNell-1 injection in a rat posterolateral spinal fusion model has been tested with successful results [109].

LMP is an intracellular LIM-domain protein acting as a potent positive regulator of the osteoblast differentiation program, being able to induce the activation of BMPs and downstream signaling pathway [113, 114]. In humans, three different splice variants are transcribed from the LMP-coding gene (PDZ and LIM domain-7, PDLIM7), named LMP1, LMP2, and LMP3. Both LMP1 and LMP3 induce osteogenic differentiation of mesenchymal progenitors and pre osteoblasts *in vitro* and bone formation in diverse animal models [12, 20, 80, 89, 104–107, 110, 114–119]. Similar to Nell-1, LMPs in humans are overexpressed in calvarial tissues and cells isolated from synostosis of patients affected by sporadic synostosis, where it possibly plays a pathogenetic role [120]. LMP1 has been used successfully to induce spine fusion in rats and rabbits, upon plasmid transfection and adenoviral vector-mediated delivery, respectively [110, 113]. Adenoviral-mediated *ex vivo* transduction was also used to overexpress LMP3 in dermal fibroblast in a mouse model of paravertebral ectopic bone formation, resulting in the formation of an overwhelming new bony mass [20].

Finally, another gene therapy approach to spine fusion has been recently performed in a rabbit model, using the Hoxc-8-interacting domain of Smad1. In this case, *ex vivo* transduction was performed using an adenoviral vector, modified to target specifically BM-MSC in order to improve the efficiency of gene transfer [83].

Overall the genetic engineering strategies proposed so far in spinal orthopedics surgery proved to be extremely effective. Much effort should be further spent in improving the safety of the gene delivery strategies by limiting the toxicity and the immunogenicity and avoiding modification that could lead to genomic instability.

## 4. Conclusions

Despite the improvement of surgical procedures, the research efforts achieved so far did not allow obtaining convincing result to suggest alternative effective methods to replace or at least flank bone grafting. Further studies and clinical trials are foreseen to achieve the goal of improving spinal surgery avoiding donor morbidity and overcoming the need for human donors.

## Figures and Tables

**Table 1:** Bone substitutes resuming.

Category	Bone substitute	Osteoinduction	Osteoconduction	Strength	Resorbability
Biological	Autografts	+	+	+	+
Biological	Allografts	+/−	+	+	+
Biological	Xenografts	+/−	+	+	+
Synthetic	Calcium-based	−	+	+/−	+/−
Synthetic	Polymer-based	−	+/−	+/−	+

+: the material has this property.

−: the material does not have this property.

+/−: the material has intermediate properties.

**Table 2:** Cell-based gene therapy in animal models of spine fusion.

Fusion site	Specie	Cell treatment	Scaffold	Reference
BM-MSC				
PF	Rat	None	Matrigel	[58]
PLF	Macaque	None	b-TCP	[59]
PLF	Goat	None	Ceramics	[60]
PLF	Rabbit	None	CRM	[61]
PLF	Mouse	None	Collagen	[62]
PLF	Rabbit	None	ProOsteon 500 R	[51]
PLF	Rat	None	Ceramic	[63]
PLF	Rabbit	None	HA/Collagen	[52]
AIBF	Pig	None	mPCL/TCP	[53]
PLF	Rat	Oxysterols	Collagen	[74]
PLF	Rabbit	None	TCP w/wo LIPUS	[75]
PF	Human	None	b-TCP	[76]
PLF	Rabbit	Hyperbaric O_2_	Alginate	[77]
PLF	Rabbit	AdBMP2	Collagen	[78]
PLF	Rabbit	AdBMP2	None	[79]
PF	Rat	AdBMP7	None	[80]
PLF	Rat	LentiBMP2 AdBMP2	Collagen	[81]
PLF	Rat	LentiBMP2	Collagen	[54]
PLF	Rabbit	rhBMP2	Alginate	[82]
PLF	Rabbit	AdSmad-1c	Gelatin	[83]

ASC				
PLF	Rat	None	TCP-Collagen	[53]
PLF	Rat	None	b-TCP-Collagen	[66]
PF	Goat	None	PLCL	[55]
PLF	Rabbit	None	nHAC-PLA	[84]
ACIF	Sheep	None	b-TCP	[85]
MLF	Pig	None	PEEK	[86]
PLF	Rat	rhBMP2	Collagen	[87]
PF	Mice	rhBMP6	None	[88]
PLF	Rat	AdBMP2	Collagen	[56]
VCF	Rat	rhBMP6	Fibrin	[89]

PF: posterior fusion; PLF: posterolateral fusion; AIBF: anterior interbody fusion; AICF: anterior interbody cervical fusion; MLF: multi-level fusion; VCF: vertebral cervical fusion; TCP: tricalcium phosphate; CRM: compression-resistant matrix; HA/Collagen: hydroxyapatite/type 1 collagen; mPCL: medical grade poly (*ε*-caprolactone); LIPUS low-intensity pulsed ultrasound, *β*-TCP: beta-tricalcium phosphate; PLCL: poly (L-lactide-co-caprolactone); nHAC-PLA: nanohydroxyapatite-collagen/polylactic acid; PEEK: polyetheretherketone.
